# CLINICAL NOTES AND CARE COORDINATION CALLS AS IMPORTANT PREDICTORS OF EMERGENCY CARE UTILIZATION IN HOME HEALTH CARE

**DOI:** 10.1093/geroni/igad104.1721

**Published:** 2023-12-21

**Authors:** Lauren Evans

**Affiliations:** VNS Health, New York City, New York, United States

## Abstract

Home care patients experience a high illness burden and a heightened risk of negative health outcomes. There is a need to understand how clinician behaviors, including notetaking and care coordination activities, can be better integrated into risk modelling in home care settings. This study predicted hospitalization/ED visits using data from 86,866 home care episodes from an agency in the Northeastern United States. We began our analysis with nearly 200 variables, including patient-related factors (e.g., comorbidities, types of medications taken, functional status), and factors relating to clinician behavior, such as problems documented in clinical notes, and calling patterns to the patient or medical care providers. We used least absolute shrinkage and selection operator (LASSO) to aid in the selection of the best subset of variables. In our preliminary analysis, we found that two clinician behavior-related factors were the strongest risk factors for experiencing hospitalization/ED visits, specifically the proportion of concerning notes generated during the care episode (OR 8.97, 95% CI 7.64, 10.52), and a measure reflecting frequent calls to the patient’s family (OR 4.51, 95% CI 3.80, 5.37). Clinician documentation of specific problems, and the clinician’s decision to conduct a medication review were also risk factors. Patient risk factors included prior hospitalization, health conditions such as cancer and renal disease), functional limitations, certain medication classes (e.g., anticoagulants, diuretics), the use of oxygen, and the use of a urinary catheter. This study demonstrates that clinician behaviors and documentation patterns can be incorporated in risk models to yield important insights.

